# Prognostic value of inflammation-based indices in patients with resected hepatocellular carcinoma

**DOI:** 10.1186/s12885-021-08153-4

**Published:** 2021-04-27

**Authors:** Weihao Kong, Mingwei Yang, Jianfeng Zhang, Ya Cheng, Tianxing Dai, Jian Zhang, Guoying Wang, Jianlin Zhang

**Affiliations:** 1 Department of Emergency Surgery, Department of Emergency Medicine, the First affiliated hospital of Anhui Medical University, 218 Jixi Avenue, Hefei, 230022 China; 2 Department of Radiation Oncology, the First affiliated hospital of Anhui Medical University, Hefei, China; 3 Department of Hepatic Surgery and Liver Transplantation Center, the Third Affiliated Hospital of Sun Yat-sen University, Guangzhou, 510630 China; 4 Organ Transplantation Institute of Sun Yat-sen University, Guangzhou, China; 5 Department of Hepatobiliary Surgery, the First Affiliated Hospital of Guangzhou Medical University, Guangzhou, China

**Keywords:** Prognosis, Liver cancer, Neutrophil-to-lymphocyte ratio, Platelet distribution width

## Abstract

**Background:**

As is well recognized that inflammation plays a crucial role in the genesis and progression of various cancer. Here we investigate the prognostic value of a novel index: the combination of neutrophil to lymphocyte ratio and platelet distribution width (coNLR-PDW) in post-operation patients with resectable hepatocellular carcinoma (HCC).

**Methods:**

The receiver operating characteristic (ROC) curve was utilized to determine the optimal cutoff values of continuous variables, including the neutrophil-lymphocyte ratio (NLR) and platelet distribution width (PDW). Kaplan-Meier method and the Log-rank test were used to compare survival differences across three groups stratified by the coNLR-PDW score. Univariate and multivariate Cox proportional hazard regression analyses were adopted to identify independent factors of HCC patient’s prognosis.

**Results:**

1.59 and 13.0 were perceived as the optimal cutoff value for NLR and PDW based on the ROC curve, respectively. Kaplan-Meier method revealed that a higher coNLR-PDW score predicts poorer overall survival (OS) and disease-free survival (DFS) (*P* < 0.001). coNLR-PDW was demonstrated as an independent factor for both OS and DFS using Cox regression analysis in training and validation cohort.

**Conclusion:**

coNLR-PDW is recognized as a valuable biomarker for predicting the survival of patients with HCC.

**Supplementary Information:**

The online version contains supplementary material available at 10.1186/s12885-021-08153-4.

## Background

Hepatocellular carcinoma (HCC) is one of the most common malignant tumors in the world, the second most common cause of cancer-related deaths, the fifth most common cancer in the world, and its incidence is increasing year by year [1]. Up to now, hepatectomy is one of the best treatments for resectable HCC, but the recurrence rate after surgical resection is still high, and the overall prognosis is not optimistic. Although several biomarkers have been identified as significantly associated with the prognosis of HCC, their predictive value is sometimes unsatisfactory due to a variety of reasons. Therefore, a useful and straightforward biomarker for determining early recurrence and prognosis of HCC has important clinical implications.

In recent years, researchers have found that systemic inflammatory responses play an essential role in the progression of cancer [2, 3]. Prognostic scoring systems based on systemic inflammatory responses have been demonstrated to be effective in many studies [4–6]. For example, Glasgow Prognostic Score (GPS), platelet to lymphocyte ratio (PLR), neutrophil-lymphocyte ratio (NLR), and monocyte to lymphocyte ratio (MLR) can help predict the prognosis of cancer patients. Besides, platelets play a crucial role in the progression and metastasis of cancer. Activated platelets can promote tumor cell proliferation, invasion, and angiogenesis. Elevated platelet counts are significantly associated with poor prognosis in numerous cancers, including vulvar squamous cell cancer, lung cancer, renal cell carcinoma, pancreatic cancer, ovarian cancer, and colorectal cancer [7–13]. Nevertheless, the total number of platelets depends on the consumption and production of platelets. Normal platelet counts may conceal the compensatory mechanisms of tumor pro-coagulation and pro-inflammatory phenotypes. Platelet distribution width (PDW), as an essential parameter for activated platelets, plays a vital role in a variety of tumors, including gastric cancer, melanoma, breast cancer, lung cancer, nasopharyngeal cancer, laryngeal cancer [14–18].

The score based on the systemic inflammatory response may reflect the patient’s systemic inflammatory response status, which may affect the prognosis of patients with HCC. Therefore, in this study, we explored the prognostic role of the coNLR-PDW score in patients with resectable HCC.

## Methods

### Patients

We conducted a retrospective review of 250 patients with HCC who underwent hepatectomy at the Third Affiliated Hospital of Sun Yat-sen University from January 2009 to October 2015. The inclusion criteria were as follows: (1) the patient underwent radical resection of HCC, (2) no chemotherapy or radiotherapy before surgery, (3) no extrahepatic metastasis, (4) HCC was confirmed by postoperative pathology, (5) negative surgical margin, (6) no infectious or other inflammatory diseases (including acute pancreatitis, acute cholecystitis, arthritis, acute hepatitis, glomerulonephritis, central nervous system infection, and so on) occurred one month before surgery. Exclusion criteria are as follows: (1) preoperative severe systemic diseases, such as hematopoietic diseases, accompanying other malignant tumors, inflammation-related diseases, etc. (2) receiving anti-tumor treatment (3) being lack of complete clinicopathological features. The stage of HCC patients is determined by the Barcelona Clinic Liver Cancer (BCLC) stage.

### Follow-up

Informed consent was obtained from all patients participating in the study, and the study was approved by the Ethics Committee of the Third Affiliated Hospital of Sun Yat-sen University and the First affiliated hospital of Anhui Medical University. After surgical resection, patients were followed up every three months for the first three years, and every six months afterward. The follow-up examination included tumor markers, abdominal ultrasound, thoracoabdominal computed tomography (CT), or magnetic resonance imaging (MRI). A bone scan was adopted when HCC recurrence was suspected. The overall survival (OS) time is defined as the interval from surgical resection to the last follow-up or death, and disease-free survival (DFS) time is defined as the interval from surgical resection to the last follow-up or disease progression. The last follow-up time was until December 2016.

### NLR-PDW evaluation

**Fig. 1:**
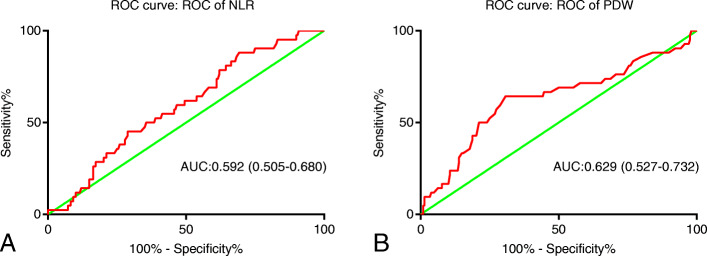
Receiver operating characteristic curve analysis of NLR and PDW in training cohort. **a**: NLR is represented by the blue line, the optimal cutoff value is 1.59, and the area under the curve (AUC) is 0.592. **b**: PDW is represented by the blue line. Similarly, the optimal cutoff value is 13.0, and the AUC is 0.629. Notes: NLR: Neutrophil to lymphocyte ratio; PDW: Platelet distribution width

We collected hematologic parameters within one week before surgery. The neutrophil count divided by the lymphocyte count is defined as NLR. The receiver operating characteristic (ROC) curve is employed to determine the optimal cutoff values for prothrombin time (PT), alanine transaminase (ALT), total bilirubin (TB), glutamyl transpeptidase (GGT), albumin (ALB), mean platelet volume (MPV), PDW, and NLR, respectively. According to the ROC curve analysis, we determined that the optimal cutoff value of NLR is 1.59, and the area under the ROC curve is 0.592. Similarly, 13.0 was identified as the optimal cutoff value of PDW, and the area under the ROC curve is 0.629 (Fig. 1).

Based on the cutoff values of NLR and PDW, we divided the patients into high NLR group (≥1.59) and low NLR group (< 1.59), high PDW group (≥13.0), and low PDW group (< 13.0), respectively. The novel scoring system for coNLR-PDW is defined as follows: patients with high NLR values and high PDW values are defined as 2, patients with low NLR values and low PDW values are defined as 0, and patients with either high indicator are defined as 1.

### Statistical analysis

We use SPSS 21.0 version (SPSS Inc., Chicago, IL, USA) and GraphPad Prism7 for statistical analysis. The ROC curve is used to evaluate the predictive value of indicators for the prognosis of HCC patients. The larger the area under the ROC curve, the better its ability to predict the survival outcome. Differences among coNLR-PDW groups were compared using the chi-squared test. The Kaplan-Meier curve is used to intuitively describe the survival status and survival time between different coNLR-PLR score groups, and the differences between them are analyzed by the Log-rank test. Univariate and multivariate Cox regression analyses were carried out to screen for independent factors of prognosis by using the stepwise method. A *p*-value below 0.05 was deemed as statistical significance.

## Results

### Patient demographics

We included a total of 250 HCC patients who underwent radical hepatectomy at the Third Affiliated Hospital of Sun Yat-sen University, including 225 men and 25 women. Of all patients, 221 patients were positive for hepatitis B surface antigen (HBsAg), and 29 patients were negative for HBsAg. There was only one lesion in 179 patients and multiple lesions in 71 patients. Based on the BCLC stage, there were 19, 79, 48, and 104 patients in stages 0, A, B, and C, respectively. The average age at diagnosis was 50 years. Detailed information is displayed in Table 1. The detailed flow chart is shown in Supplementary Figure 1.

**Table 1:** Correlation between coNLR-PDW and clinicopathological features or hematological parameters

Variables	Number of patients	co NLR-PDW 0	co NLR-PDW 1	co NLR-PDW 2	P-value
Age, < 60/≥60 years	194/56	31/11	108/35	55/10	0.281
Gender, male/female	225/25	36/6	129/14	60/5	0.536
HBsAg, negative/positive	29/221	5/37	17/126	7/58	0.971
Cirrhosis, no/yes	79/171	21/21	42/99	16/44	**0.004**
Tumor size, ≤5/> 5 cm	137/113	31/11	69/74	37/28	**0.013**
Tumor number, single/multiple	179/71	32/10	99/44	48/17	0.609
AFP, < 400/≥400 ng/ml	170/80	26/16	98/45	46/19	0.617
Vascular invasion, no/yes	146/104	28/14	77/66	41/24	0.225
Child-Pugh grade, A/B	235/15	41/1	137/6	57/8	**0.041**
BCLC, 0-A/ B-C	19/79/48/104	23/19	50/93	25/40	0.069
Differentiation, well moderate/poor	41/189/20	40/2	130/13	60/5	0.658
PT (s)	13.6 (12.9–14.2)	13.4 (12.7–14.1)	13.6 (13.0–14.4)	13.5 (13.1–14.2)	0.143
ALT (U/L)	38.0 (26.0–52.3)	35.0 (22.5–46.5)	40.0 (27.8–57.5)	39.0 (26.0–50.0)	0.093
TB (umol/L)	14.7 (10.7–18.3)	13.9 (9.8–16.0)	14.3 (10.8–18.2)	15.8 (12.4–20.5)	**0.032**
GGT (U/L)	60.5 (37.0–117.5)	49.0 (26.0–98.0)	64.5 (38.0–119.5)	70.0 (43.0–135.0)	0.055
ALB (g/L)	40.0 ± 4.2	40.3 ± 3.7	39.5 ± 4.1	41.0 ± 4.6	0.053
MPV (Fl)	10.6 (9.9–11.2)	9.8 (9.4–10.2)	10.6 (10.0–11.2)	11.2 (10.8–11.6)	**< 0.001**

Notes: *AFP* alpha fetoprotein, *BCLC* Barcelona Clinic Liver Cancer, *PT* prothrombin time, *ALT* Alanine transaminase, *TB* total bilirubin, *GGT* gamma-glutamyl transpeptidase, *ALB* albumin, *MPV* mean platelet volume, *coNLR-PDW* combination of NLR and PDW

### Correlation between the clinicopathologic features or hematologic parameters and coNLR-PDW

The correlation between coNLR-PDW and clinicopathological features or hematological parameters is shown In Table 1. Significant correlation were found in cirrhosis (*P* = 0.004), tumor size (*P* = 0.013), Child-Pugh grade (*P* = 0.041), TB (*P* = 0.032), and MPV (*P* < 0.001) among three coNLR-PDW groups. However, there were no statistical significance in age, gender, HBsAg, tumor number, alpha-fetoprotein (AFP), vascular invasion, BCLC stage, differentiation, PT, ALT, GGT, and ALB.

### Survival analysis of coNLR-PDW

**Fig. 2:**
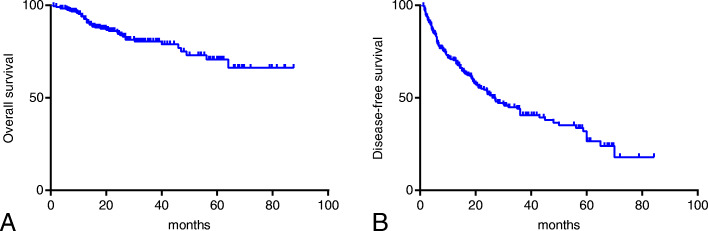
OS and DFS curves for patients with whole resectable HCC patients. **a**: Overall survival curves of the whole cohort. **b**: Disease-free survival curves of the whole cohort. Notes: OS: overall survival; DFS: disease-free survival; HCC: hepatocellular carcinoma

**Fig. 3:**
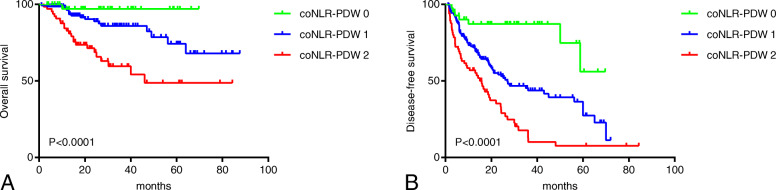
OS and DFS curves stratified by coNLR-PDW score in training HCC cohort. **a**: Patients with higher coNLR-PDW score had poorer OS (*P* < 0.0001). **b**: Patients with higher coNLR-PDW score had poorer DFS (P < 0.0001). Notes: OS: overall survival; DFS: disease-free survival; HCC: hepatocellular carcinoma

**Fig. 4:**
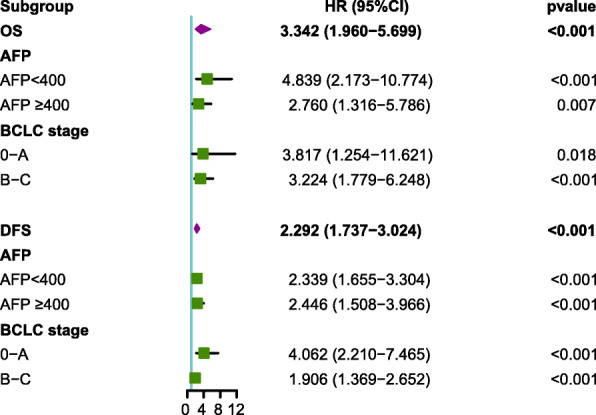
OS and DFS curves stratified by coNLR-PDW for AFP and BCLC stage subgroup in training HCC cohort. Notes: OS: overall survival; DFS: disease-free survival; NLR: Neutrophil to lymphocyte ratio; PDW: Platelet distribution width; AFP: alpha fetoprotein; BCLC: Barcelona Clinic Liver Cancer; HCC: hepatocellular carcinoma

OS and DFS curves for patients with whole resectable HCC were shown in Fig. 2. Kaplan-Meier analysis and log-rank test were adopted to identify the survival differences among the three groups stratified by the coNLR-PDW score. The OS curves of the three groups separate significantly, patients with coNLR-PDW scoring 2 has the worst survival compared with those with coNLR-PDW scoring 1 and 0. Similarly, the DFS curves classified by the coNLR-PDW score shows that DFS duration with coNLR-PDW scoring 0 is superior to that of co-NLR-PDW scoring 1, and coNLR-PDW scoring 1 is better than coNLR-PDW scoring 2 in DFS (Fig. 3). Additionally, further analysis was performed in the subgroups of AFP (<400 ng/ml and ≥ 400 ng/ml) and BCLC stage (0-A and B-C). Higher score of coNLR-PDW predicted worse OS and DFS in the AFP subgroup (AFP<400 ng/ml: *P* < 0.001 for OS, P < 0.001 for DFS; AFP ≥400 ng/ml: *P* = 0.007 for OS, P < 0.001 for DFS), and BCLC stage (BCLC 0-A: *P* = 0.018 for OS, P < 0.001 for DFS; BCLC B-C: P < 0.001 for OS, P < 0.001 for DFS) (Fig. 4).

### Univariate and multivariate analyses for OS

According to univariate regression analyses, significant correlation was observed between tumor size (HR: 2.664, 95%CI: 1.424–4.983, *P* = 0.002), AFP (HR: 2.310, 95%CI: 1.253–4.258, *P* = 0.007), vascular invasion (HR: 2.327, 95%CI: 1.261–4.293, P = 0.007), BCLC stage (HR: 2.543, 95%CI: 1.248–5.181, *P* = 0.010), ALT (HR: 2.082, 95%CI: 1.046–4.146, *P* = 0.037), GGT (HR: 3.825, 95%CI: 1.697–8.626, *P* = 0.001), coNLR-PDW (HR: 3.342, 95%CI: 1.960–5.699, *P* < 0.001) and OS. Then these identified factors were selected for multivariate regression analysis, AFP (HR: 2.782, 95%CI: 1.496–5.173, P = 0.001), GGT (HR: 3.852, 95%CI: 1.690–8.782, P = 0.001), and coNLR-PDW (HR: 3.721, 95%CI: 2.150–6.440, *P* < 0.001) correlated with unfavorable OS significantly (Table 2).

**Table 2:** Univariate and multivariate analyses for OS in training cohort

Variable	Univariate analysis			Multivariate analysis		
	p	Hazard ratio	95%confidence interval	p	Hazard ratio	95%confidence interval
Age (years)(≥60 versus < 60)	0.066	0.380	0.136–1.065			
Gender (female versus male)	0.783	0.865	0.308–2.431			
HBsAg (yes versus no)	0.184	2.621	0.633–10.850			
Cirrhosis (yes versus no)	0.652	1.153	0.621–2.140			
Tumor size(> 5 versus ≤5)	**0.002**	**2.664**	**1.424–4.983**	**–**		
Tumor number (multiple versus single)	0.087	1.726	0.924–3.223			
AFP(≥400 versus < 400)	**0.007**	**2.310**	**1.253–4.258**	**0.001**	**2.782**	**1.496–5.173**
Vascular invasion (yes versus no)	**0.007**	**2.327**	**1.261–4.293**	**–**		
Child-Pugh grade (B versus A)	0.351	0.389	0.053–2.828			
BCLC Stage(B + C versus 0 + A)	**0.010**	**2.543**	**1.248–5.181**	**–**		
Differentiation (poor versus well/moderate)	0.285	1.666	0.653–4.247			
PT (≥13.7 versus < 13.7)	0.094	0.578	0.304–1.099			
ALT (≥ 34 versus < 34)	**0.037**	**2.082**	**1.046–4.146**	–		
TB (≥12.5 versus < 12.5)	0.230	0.688	0.373–1.268			
GGT (≥49 versus < 49)	**0.001**	**3.825**	**1.697–8.626**	**0.001**	**3.852**	**1.690–8.782**
ALB (≥39.0 versus < 39.0)	0.201	1.548	0.792–3.028			
MPV (≥11.2 versus < 11.2)	0.424	1.300	0.684–2.470			
co NLR-PDW	**< 0.001**	**3.342**	**1.960–5.699**	**< 0.001**	**3.721**	**2.150–6.440**

Notes: *AFP* alpha fetoprotein, *BCLC* Barcelona Clinic Liver Cancer, *PT* prothrombin time, *ALT* Alanine transaminase, *TB* total bilirubin, *GGT* gamma-glutamyl transpeptidase, *ALB* albumin, *MPV* mean platelet volume, *coNLR-PDW* combination of NLR and PDW, *OS* overall survival

### Univariate and multivariate analyses for DFS

Tumor size (HR: 2.332, 95%CI: 1.650–3.296, *P* < 0.001), Tumor number (HR: 2.198, 95%CI: 1.551–3.114, P < 0.001), AFP (HR: 1.452, 95%CI: 1.010–2.087, *P* = 0.044), vascular invasion (HR: 2.095, 95%CI: 1.485–2.955, P < 0.001), BCLC stage (HR: 2.893, 95%CI: 1.932–4.332, P < 0.001), GGT (HR: 2.084, 95%CI: 1.431–3.036, *P* < 0.001), and coNLR-PDW (HR: 2.292, 95%CI: 1.737–3.024, P < 0.001) are significantly associated with DFS according to univariate regression analysis. Further multivariate regression analysis displays that tumor number (HR: 1.599, 95%CI: 1.101–2.323, *P* = 0.014), AFP (HR: 1.471, 95%CI: 1.014–2.135, *P* = 0.042), BCLC stage (HR: 2.078, 95%CI: 1.333–3.238, *P* = 0.001), GGT (HR: 1.714, 95%CI: 1.163–2.526, *P* = 0.006), and coNLR-PDW (HR: 2.370, 95%CI: 1.781–3.152, P < 0.001) are independent prognostic factors for DFS (Table 3).

**Table 3:** Univariate and multivariate analyses for DFS in training cohort

Variable	Univariate analysis			Multivariate analysis		
	p	Hazard ratio	95%confidence interval	p	Hazard ratio	95%confidence interval
Age (years)(≥60 versus < 60)	0.355	0.819	0.537–1.250			
Gender (female versus male)	0.437	0.783	0.422–1.452			
HBsAg (yes versus no)	0.345	1.306	0.750–2.274			
Cirrhosis (yes versus no)	0.405	1.154	0.823–1.618			
Tumor size (> 5 versus ≤5)	**< 0.001**	**2.332**	**1.650–3.296**	**–**		
Tumor number (multiple versus single)	**< 0.001**	**2.198**	**1.551–3.114**	**0.014**	**1.599**	**1.101–2.323**
AFP(≥400 versus < 400)	**0.044**	**1.452**	**1.010–2.087**	**0.042**	**1.471**	**1.014–2.135**
Vascular invasion (yes versus no)	**< 0.001**	**2.095**	**1.485–2.955**	**–**		
Child-Pugh grade (B versus A)	0.067	1.746	0.963–3.166			
BCLC Stage(B + C versus 0 + A)	**< 0.001**	**2.893**	**1.932–4.332**	**0.001**	**2.078**	**1.333–3.238**
Differentiation (poor versus well/moderate)	0.112	1.568	0.900–2.730			
PT (≥13.7 versus < 13.7)	0.326	0.841	0.595–1.188			
ALT (≥ 34 versus < 34)	0.100	1.347	0.944–1.920			
TB (≥12.5 versus < 12.5)	0.889	1.026	0.719–1.463			
GGT (≥49 versus < 49)	**< 0.001**	**2.084**	**1.431–3.036**	**0.006**	**1.714**	**1.163–2.526**
ALB (≥39.0 versus < 39.0)	0.437	0.871	0.616–1.233			
MPV (≥11.2 versus < 11.2)	0.228	1.257	0.867–1.822			
co NLR-PDW	**< 0.001**	**2.292**	**1.737–3.024**	**< 0.001**	**2.370**	**1.781–3.152**

Notes: *AFP* alpha fetoprotein, *BCLC* Barcelona Clinic Liver Cancer, *PT* prothrombin time, *ALT* Alanine transaminase, *TB* total bilirubin, *GGT* gamma-glutamyl transpeptidase, *ALB* albumin, *MPV* mean platelet volume, *coNLR-PDW* combination of NLR and PDW, *DFS* disease free survival

### Comparison of the predictive value among different combinations

**Fig. 5:**
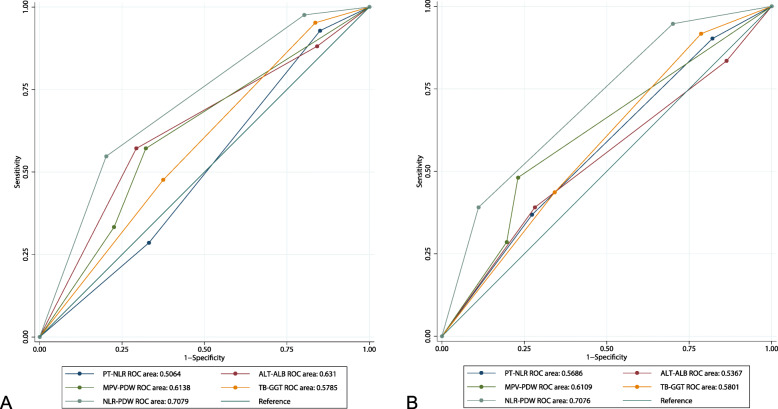
Comparison of the predictive value among different combinations for OS (**a**) and DFS (**b**) in training cohort. Notes: OS: overall survival; DFS: disease-free survival

ROC curve is used to evaluate the predictive value of different combined indicators for OS and DFS. In the training cohort, the predictive value of coNLR-PDW (AUC for OS: 0.7079; AUC for DFS: 0.7076) is better than other joint indicators for predicting OS and DFS (Fig. 5).

### External validation

We also included 91 HCC patients from the First Affiliated Hospital of Anhui Medical University for external verification. The results of the survival analysis showed that HCC patients with a coNLR-PDW score of 2 had worse OS and DFS when compared with the other two groups (supplementary Figure 2). Meanwhile, the results of univariate Cox regression analysis showed that tumor size, tumor number, BCLC stage, and coNLR-PDW were significantly related to OS and DFS. Furthermore, the results of multivariate Cox regression analysis indicate that coNLR-PDW is an independent risk factor for OS and DFS in HCC patients (supplementary Table 1–2). The predictive value of coNLR-PDW (AUC for OS: 0.7662; AUC for DFS: 0.7448) is better than other joint indicators for predicting OS and DFS (supplementary Figure 3).

## Discussion

HCC ranks the seventh of the newly diagnosed malignancies, and it accounts for 8.2% of the cancer death according to cancer statistics [19]. The main reason for high mortality lies in the intrinsic pathophysiological characteristics of liver cancer, as well as the lack of early diagnosis and effective treatment. Up to date, the best treatment for resectable HCC is hepatectomy. However, the rate of recurrence and metastasis after radical hepatectomy is still high. Therefore, it is imperative to explore practical and readily available biomarker to provide prognostic information, and thus instruct an optimal treatment strategy.

Since Virchow first introduced the correlation between inflammation and malignancies [20], mounting evidence has confirmed that inflammation influence every step of tumor development and tumor promotion [21]. Systemic Inflammatory response became a hallmark of cancer and was demonstrated to correlate with survivals of various carcinomas. HCC is one of inflammation-related cancer for infections with hepatitis B, or C viruses increase the risk of HCC, especially in China, where there is a high rate of hepatitis B infection. Moreover, accumulated evidence has demonstrated the association between inflammation-based indices and the prognosis of hepatocellular cancer [22].

NLR has been extensively investigated as the prognostic value in multiple cancers, including HCC [5]. The mechanisms by which NLR predict prognosis remains unclear. Possible interpretation including that neutrophils exert tumor-promoting effect by the production of cytokines, proteases and reactive oxygen species (ROS), and inhibition of the cytolytic activity of immune cells, such as lymphocytes, activated T cells, and natural killer cells [21, 23]. Lymphocytes play a crucial role in anti-tumor immune activity. A high level of lymphocyte was revealed to correlate with favorable survival in multiple cancers [24–26]. In our study, the cutoff value for NLR is 1.58. High NLR was recognized as an independent indicator for unfavorable OS and DFS in univariate analysis. The result is in agreement with the previous studies [23].

Platelets were revealed to activate tumor progression by secreting cellular growth factors such as platelet-derived growth factor (PDGF), vascular endothelial growth factor (VEGF) [27]. Moreover, it is reported platelets-cancer interactions activate tumor angiogenesis, progression, and metastasis [28]. Thrombocytosis is revealed to correlate with poor survival of multiple cancers [29]. Nevertheless, platelet activity is not only determined by platelet count, but also platelet size. A large platelet was considered active and capable of more granules. PDW, an index that reveals the variation in platelet size, was founded to reflect platelet activity comprehensively [30]. Additionally, PDW was also confirmed to be associated with cancer prognosis in several cancers [14–18]. Currently, a significant association was detected between PDW and survival, including both OS and DFS, which is in accordance with previous studies.

Elevated NLR and PDW may reveal the imbalance tilted toward pro-tumor inflammation. coNLR-PDW index, proposed in Song’s study, had been demonstrated to be adversely associated with survival of non-metastatic colorectal cancer [31]. Compared with NLR or PDW, coNLR-PDW is considered more comprehensive and sensitive. To our knowledge, coNLR-PDW has not yet been explored in HCC. In the current study, significant relationships were revealed between coNLR-PDW groups and clinicopathologic characteristics, such as cirrhosis, tumor size, and Child-Pugh grade, which show consistency between coNLR-PDW score and tumor progression. Furthermore, as indicated by Kaplan-Meier analysis and log-rank test, coNLR-PDW can be utilized to predict survival for resectable HCC patients. The higher coNLR-PDW score predicts more inferior OS and DFS. The coNLR-PDW score was recognized as independent prognostic markers for both OS and DFS of resectable HCC patients according to the univariate and multivariate analysis. Finally, we also validate our results using an external HCC cohort. Taken these into consideration, the coNLR-PDW score is considered as a promising prognostic biomarker in resectable hepatocellular carcinoma.

We have to admit that there exist several limitations in our study. First, this is a retrospective study, so a potential bias is inevitable. Second, the inflammatory indicators are not only affected by tumors but also affected by other factors, such as geographical and ethnic differences. Third, the study was mainly carried out on a hepatitis B population, and the proposed novel score may not apply to the HCC population having other etiology in the background. Lastly, some biomarkers, such as CRP, were not routinely tested in our center. Thus, they were not included in the analysis.

## Conclusion

CoNLR-PDW, which bears a significant association with survival, can serve as a valuable and useful biomarker for resectable HCC.

## Supplementary Information

**Additional file 1: Supplementary Figure 1.** The detailed flow chart of this research.

**Additional file 2: Supplementary Figure 2.** OS (A) and DFS (B) curves stratified by coNLR-PDW score in validation HCC cohort. Notes: OS: overall survival; DFS: disease-free survival; HCC: hepatocellular carcinoma.

**Additional file 3: Supplementary Figure 3.** Comparison of the predictive value among different combinations for OS (A) and DFS (B) in validation cohort. Notes: OS: overall survival; DFS: disease-free survival.

**Additional file 4: Supplementary Table 1.** Univariate and multivariate analyses for OS in validation cohort. Notes: AFP: alpha fetoprotein; BCLC: Barcelona Clinic Liver Cancer; PT: prothrombin time; ALT:Alanine transaminase; TB: total bilirubin; GGT: gamma-glutamyl transpeptidase; ALB: albumin; MPV: mean platelet volume; coNLR-PDW: combination of NLR and PDW; OS: overall survival. **Supplementary Table 2**. Univariate and multivariate analyses for DFS in validation cohort. Notes: AFP: alpha fetoprotein; BCLC: Barcelona Clinic Liver Cancer; PT: prothrombin time; ALT: Alanine transaminase; TB: total bilirubin; GGT: gamma-glutamyl transpeptidase; ALB: albumin; MPV: mean platelet volume; coNLR-PDW: combination of NLR and PDW; DFS: disease free survival.

## Data Availability

Data used to support the results of this study can be obtained from the corresponding author.

## Abbreviations

HCC: Hepatocellular carcinoma
ROC: Receiver operating characteristic
NLR: Neutrophil-lymphocyte ratio
PDW: Platelet distribution width
OS: Overall survival
DFS: Disease-free survival
GPS: Glasgow Prognostic Score
PLR: Platelet to lymphocyte ratio
MLR: Monocyte to lymphocyte ratio
CT: Computed tomography
MRI: Magnetic resonance imaging
PT: Prothrombin time
ALT: Alanine transaminase
TB: Total bilirubin
GGT: Glutamyl transpeptidase
ALB: Albumin
AFP: Alpha-fetoprotein
ROS: Reactive oxygen species
PDGF: Platelet-derived growth factor
VEGF: Vascular endothelial growth factor
