# Supplementary figures and images for: Prognostic value of inflammation-based indices in patients with resected hepatocellular carcinoma
Source: BMC Cancer. 2021 Apr 27;21:469. doi: 10.1186/s12885-021-08153-4 (PMC8077869; doi:10.1186/s12885-021-08153-4)

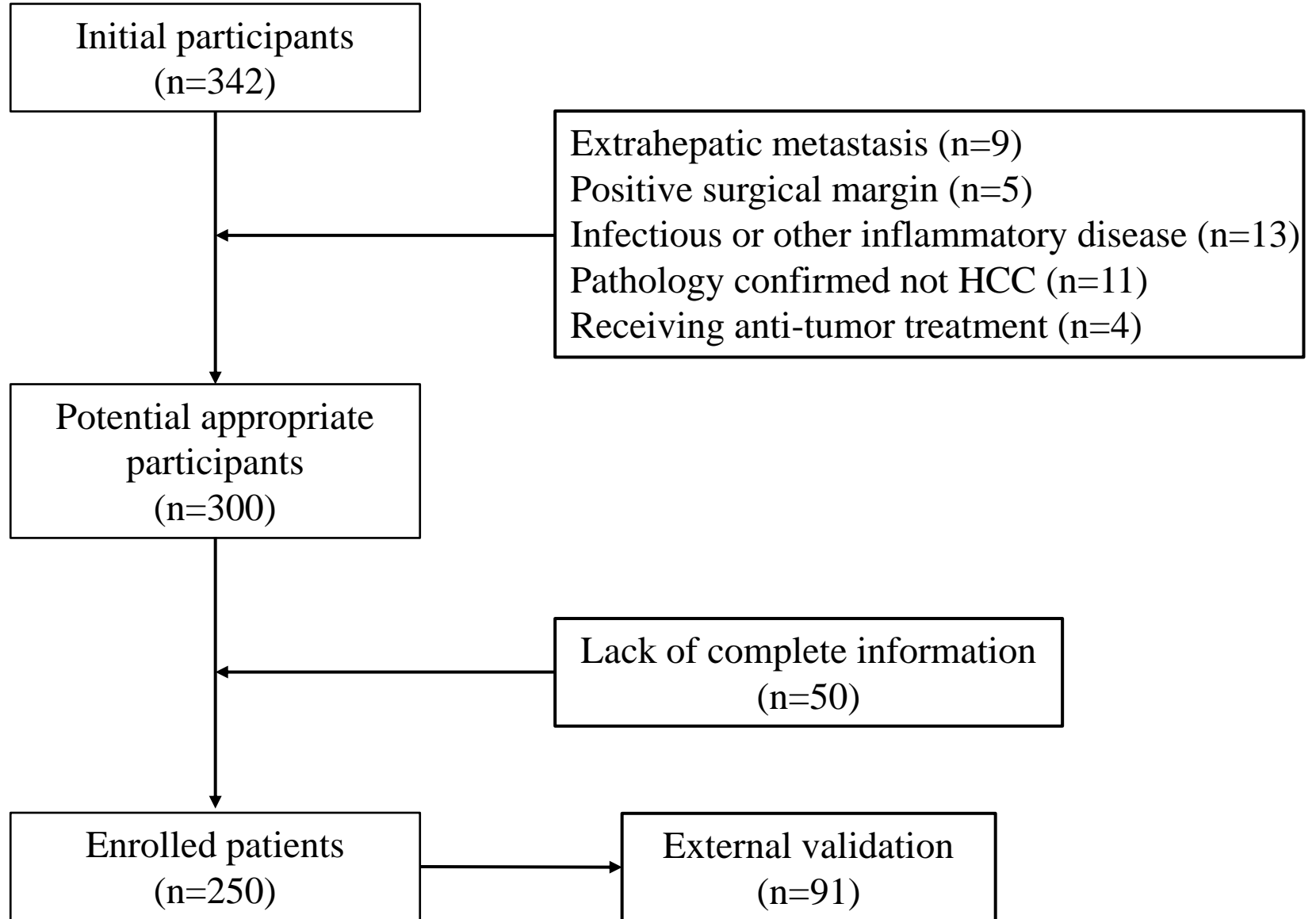

Supplement: Supplementary file 1 — Additional file 1: Supplementary Figure 1. The detailed flow chart of this research. [file 12885_2021_8153_MOESM1_ESM.pdf]

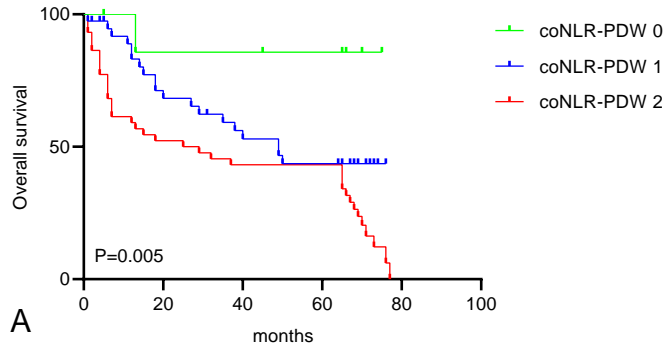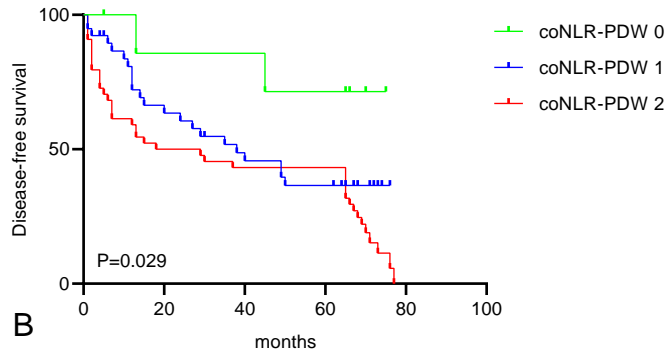

Supplement: Supplementary file 2 — Additional file 2: Supplementary Figure 2. OS (A) and DFS (B) curves stratified by coNLR-PDW score in validation HCC cohort. Notes: OS: overall survival; DFS: disease-free survival; HCC: hepatocellular carcinoma. [file 12885_2021_8153_MOESM2_ESM.pdf]

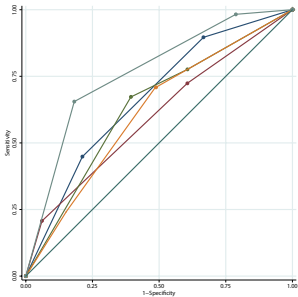

A

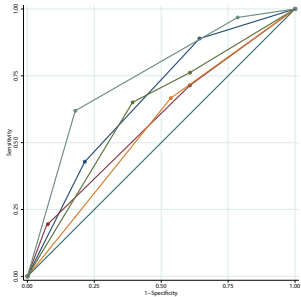

B

Supplement: Supplementary file 3 — Additional file 3: Supplementary Figure 3. Comparison of the predictive value among different combinations for OS (A) and DFS (B) in validation cohort. Notes: OS: overall survival; DFS: disease-free survival. [file 12885_2021_8153_MOESM3_ESM.pdf]
