# Supplementary material for: Prognostic value of inflammation-based indices in patients with resected hepatocellular carcinoma
Source: BMC Cancer. 2021 Apr 27;21:469. doi: 10.1186/s12885-021-08153-4 (PMC8077869; doi:10.1186/s12885-021-08153-4)
Supplement: Supplementary file 4 — Additional file 4: Supplementary Table 1. Univariate and multivariate analyses for OS in validation cohort. Notes: AFP: alpha fetoprotein; BCLC: Barcelona Clinic Liver Cancer; PT: prothrombin time; ALT:Alanine transaminase; TB: total bilirubin; GGT: gamma-glutamyl transpeptidase; ALB: albumin; MPV: mean platelet volume; coNLR-PDW: combination of NLR and PDW; OS: overall survival. Supplementary Table 2. Univariate and multivariate analyses for DFS in validation cohort. Notes: AFP: alpha fetoprotein; BCLC: Barcelona Clinic Liver Cancer; PT: prothrombin time; ALT: Alanine transaminase; TB: total bilirubin; GGT: gamma-glutamyl transpeptidase; ALB: albumin; MPV: mean platelet volume; coNLR-PDW: combination of NLR and PDW; DFS: disease free survival. [file 12885_2021_8153_MOESM4_ESM.docx]

**Supplementary Table1**. Univariate and multivariate analyses for OS in validation cohort

| Variable | Univariate analysis | | | Multivariate analysis | | |
| --- | --- | --- | --- | --- | --- | --- |
|  | p | Hazard ratio | 95%confidence interval | p | Hazard ratio | 95%confidence interval |
| Age（years)(≥60 versus <60) | 0.400 | 1.253 | 0.741-2.117 |  |  |  |
| Gender (female versus male) | 0.940 | 1.030 | 0.485-2.184 |  |  |  |
| HBsAg (yes versus no) | 0.939 | 1.024 | 0.550-1.907 |  |  |  |
| Cirrhosis (yes versus no) | 0.561 | 0.836 | 0.457-1.530 |  |  |  |
| Tumor size(>5 versus ≤5） | **0.010** | **2.057** | **1.191-3.552** | **-** |  |  |
| Tumor number (multiple versus single) | **<0.001** | **4.459** | **2.472-8.044** | **<0.001** | **3.495** | **1.881-6.493** |
| AFP(≥400 versus <400） | 0.309 | 1.341 | 0.762-2.361 |  |  |  |
| Vascular invasion (yes versus no) | 0.503 | 1.337 | 0.572-3.126 |  |  |  |
| Child-Pugh grade (B versus A) | 0.442 | 1.742 | 0.423-7.174 |  |  |  |
| BCLC Stage(B+C versus 0+A） | **<0.001** | **3.029** | **1.624-5.652** | **0.040** | **2.012** | **1.033-3.921** |
| Differentiation(poor versus well/moderate） | 0.117 | 1.702 | 0.876-3.306 |  |  |  |
| PT (≥13.7 versus <13.7） | 0.624 | 1.139 | 0.677-1.917 |  |  |  |
| ALT (≥ 34 versus <34） | 0.144 | 0.671 | 0.393-1.146 |  |  |  |
| TB (≥12.5 versus <12.5） | 0.179 | 0.700 | 0.416-1.177 |  |  |  |
| GGT (≥49 versus <49） | 0.729 | 0.911 | 0.536-1.547 |  |  |  |
| ALB (≥39.0 versus <39.0） | 0.185 | 0.688 | 0.395-1.197 |  |  |  |
| MPV (≥11.2 versus <11.2） | 0.213 | 1.422 | 0.817-2.475 |  |  |  |
| co NLR-PDW | **0.002** | **2.131** | **1.329-3.417** | **0.003** | **2.064** | **1.275-3.341** |

Notes: AFP : alpha fetoprotein; BCLC : Barcelona Clinic Liver Cancer; PT : prothrombin time; ALT：Alanine transaminase; TB: total bilirubin; GGT: gamma-glutamyl transpeptidase; ALB: albumin; MPV: mean platelet volume; coNLR-PDW: combination of NLR and PDW; OS: overall survival.

**Supplementary Table2**. Univariate and multivariate analyses for DFS in validation cohort

| Variable | Univariate analysis | | | Multivariate analysis | | |
| --- | --- | --- | --- | --- | --- | --- |
|  | p | Hazard ratio | 95%confidence interval | p | Hazard ratio | 95%confidence interval |
| Age（years)(≥60 versus <60) | 0.549 | 1.166 | 0.705-1.930 |  |  |  |
| Gender (female versus male) | 0.762 | 0.891 | 0.422-1.880 |  |  |  |
| HBsAg (yes versus no) | 0.861 | 1.055 | 0.580-1.918 |  |  |  |
| Cirrhosis (yes versus no) | 0.683 | 0.886 | 0.495-1.586 |  |  |  |
| Tumor size (>5 versus ≤5） | **0.035** | **1.735** | **1.039-2.896** | **-** |  |  |
| Tumor number (multiple versus single) | **<0.001** | **3.537** | **1.999-6.257** | **<0.001** | **3.529** | **1.990-6.259** |
| AFP(≥400 versus <400） | 0.323 | 1.317 | 0.763-2.273 |  |  |  |
| Vascular invasion (yes versus no) | 0.582 | 1.267 | 0.545-2.950 |  |  |  |
| Child-Pugh grade (B versus A) | 0.556 | 1.529 | 0.372-6.280 |  |  |  |
| BCLC Stage(B+C versus 0+A） | **0.004** | **2.277** | **1.299-3.994** | **-** |  |  |
| Differentiation (poor versus well/moderate） | 0.250 | 1.470 | 0.762-2.836 |  |  |  |
| PT (≥13.7 versus <13.7） | 0.424 | 1.226 | 0.744-2.019 |  |  |  |
| ALT (≥ 34 versus <34） | 0.147 | 0.684 | 0.410-1.142 |  |  |  |
| TB (≥12.5 versus <12.5） | 0.597 | 0.874 | 0.530-1.441 |  |  |  |
| GGT (≥49 versus <49） | 0.879 | 1.041 | 0.624-1.735 |  |  |  |
| ALB (≥39.0 versus <39.0） | 0.487 | 0.825 | 0.479-1.420 |  |  |  |
| MPV (≥11.2 versus <11.2） | 0.381 | 1.264 | 0.748-2.133 |  |  |  |
| co NLR-PDW | **0.010** | **1.743** | **1.140-2.665** | **0.011** | **1.759** | **1.138-2.719** |

Notes: AFP : alpha fetoprotein; BCLC : Barcelona Clinic Liver Cancer; PT : prothrombin time; ALT：Alanine transaminase; TB: total bilirubin; GGT: gamma-glutamyl transpeptidase; ALB: albumin; MPV: mean platelet volume; coNLR-PDW: combination of NLR and PDW; DFS: disease free survival.
